# iPSC-Derived Macrophages: The Differentiation Protocol Affects Cell Immune Characteristics and Differentiation Trajectories

**DOI:** 10.3390/ijms232416087

**Published:** 2022-12-16

**Authors:** Anna Klepikova, Tatiana Nenasheva, Olga Sheveleva, Elena Protasova, Daniil Antonov, Anastasiia Gainullina, Evgeniia Chikina, Olga Sakovnich, Tatiana Gerasimova, Irina Nikitina, Dmitry Shevalie, Irina Lyadova

**Affiliations:** 1 Genomics Core Facility, Skolkovo Institute of Science and Technology, Bolshoy Boulevard 30, Bld. 1, 121205 Moscow, Russia; 2 Laboratory of Cellular and Molecular Basis of Histogenesis, Koltzov Institute of Developmental Biology of the Russian Academy of Sciences, Vavilova Str., 26, 119334 Moscow, Russia; 3 Laboratory of Cell Biology, Koltzov Institute of Developmental Biology of the Russian Academy of Sciences, Vavilova Str., 26, 119334 Moscow, Russia; 4 Laboratory of Bioinformatics and Molecular Genetics, Koltzov Institute of Developmental Biology of the Russian Academy of Sciences, Vavilova Str., 26, 119334 Moscow, Russia

**Keywords:** induced pluripotent stem cells, macrophages derived from induced pluripotent stem cells, macrophage differentiation, inflammatory response, antigen presentation, lipid homeostasis

## Abstract

The generation of human macrophages from induced pluripotent stem cells (iMacs) is a rapidly developing approach used to create disease models, screen drugs, study macrophage–pathogen interactions and develop macrophage-based cell therapy. To generate iMacs, different types of protocols have been suggested, all thought to result in the generation of similar iMac populations. However, direct comparison of iMacs generated using different protocols has not been performed. We have compared the productivity, the differentiation trajectories and the characteristics of iMacs generated using two widely used protocols: one based on the formation of embryoid bodies and the induction of myeloid differentiation by only two cytokines, interleukin-3 and macrophage colony-stimulating factor, and the other utilizing multiple exogenous factors for iMac generation. We report inter-protocol differences in the following: (i) protocol productivity; (ii) dynamic changes in the expression of genes related to inflammation and lipid homeostasis following iMac differentiation and (iii) the transcriptomic profiles of terminally differentiated iMacs, including the expression of genes involved in inflammatory response, antigen presentation and lipid homeostasis. The results document the dependence of fine iMac characteristics on the type of differentiation protocol, which is important for further development of the field, including the development of iMac-based cell therapy.

## 1. Introduction

The generation of macrophages from induced pluripotent stem cells (iPSCs) is an attractive and rapidly developing direction of current biomedicine. iPSC-derived macrophages (iMacs) have been successfully used to model rare hereditary diseases associated with an impaired phagocyte function [1,2,3,4], to screen drugs [5,6], to study different aspects of macrophage–pathogen interactions [7,8,9,10] and to address fundamental questions of macrophage biology and differentiation [11,12,13]. Furthermore, iMacs hold great promise for cell therapy of different diseases. Genetically unmodified iMacs rescued mice from experimental infections caused by *Pseudomonas aeruginosa* and *Staphylococcus aureus* [14,15], and adoptive transfer of genetically corrected iMacs or iPSC-derived myeloid cell lines had beneficial therapeutic effects in experimental models of pulmonary alveolar proteinosis and cancer [16,17,18]. The versatility of iMac applications relies on the role that these cells play in the support of host homeostasis and disease pathogenesis, as well as on the advantages of the iMac methodology. The latter include the possibility to generate iMacs from any donor, to scale up the production of standardizable iMac populations and to obtain genetically modified iMacs [19,20].

The differentiation of iMacs from iPSCs is a stepwise process that goes through four main stages, i.e., (i) the formation of mesoderm and hemogenic endothelium (HE), (ii) the hematopoietic induction, (iii) the myeloid specification resulting in the generation of CD14^+^ monocyte-like iMac precursors and (iv) the differentiation of the latter into terminally differentiated iMacs [20]. To induce these stages, a number of protocols have been suggested. In most of them, terminal differentiation of iMacs is induced in the same way, i.e., by cultivating monocytic iMac precursors in the presence of macrophage colony-stimulating factor (M-CSF). In contrast, methodologies applied to drive the first three differentiation stages vary. Their variety may be reduced to four main types.

In OP9-dependent protocols, hematopoietic differentiation is achieved by co-culturing iPSCs with OP9 mouse bone-marrow-derived stromal cells [21]. The protocols were the first to be developed but are currently less often implemented due to the use of xenogeneic cells and poorly defined culture conditions. 

OP9-independent protocols can be categorized into three groups, i.e., embryoid-body-dependent spontaneous (EBS), embryoid-body-dependent exogenous factor assisted (EBF) and embryoid-body-independent (2D) exogenous factor-dependent (2DF) protocols [20].

In EBS protocols, iPSCs are cultured in low-adhesive conditions, which stimulate spontaneous generation of 3D structures called embryoid bodies (EBs). Within the EBs, tight inter-cellular interactions promote spontaneous formation of different germ layers, including the mesoderm. After EBs are formed, they are cultured in the presence of IL-3 and M-CSF, which induces the formation of hematopoietic progenitors and their myeloid specification [1,7,22,23,24].

In EBF protocols, EBs are also generated, but mesoderm induction is enforced by exogenous factors stimulating the formation of mesoderm and HE, primarily by bone morphogenetic protein 4 (BMP4), vascular endothelial growth factor A (VEGFA) and stem cell factor (SCF). The subsequent hematopoietic induction and myeloid specification are driven either by IL-3 and M-CSF only [22,25,26,27], or by a variety of exogenous factors (i.e., VEGFA, SCF, Fms-related receptor tyrosine kinase 3 ligand (Flt3L), thrombopoietin (TPO), and finally, IL-3 and M-CSF [3,28]).

In 2DF protocols, iPSCs are cultured in monolayers on matrix-coated surfaces, and EBs are not formed. The differentiation fully depends on multiple exogenous factors and small molecules that are sequentially added to the cultures to direct cell transition through sequential differentiation stages (e.g., BMP4, VEGFA, CHIR99021, Activin A, basic fibroblast growth factor (FGF2), SCF, TPO, M-CSF, etc.) [20,29,30].

In spite of the profound differences, all protocols result in the formation of cells displaying similar macrophage morphology (i.e., large vacuolated cells), phenotype (i.e., CD14^+^, CD11b^+^, CD45^+^ and CD18^+^) and functional activities, including phagocytosis and reactivity to inflammatory stimuli [14,22,23,31,32]. Nevertheless, the general similarity of iMacs obtained through different protocols does not prevent the cells from differing in their fine characteristics and/or the nuances of the differentiation trajectories. Understanding how culture conditions affect iMac properties is critically important for the development of future iMac applications. In this study, we have compared two “polar” iMac differentiation protocols, EBS and 2DF. We show significant inter-protocol differences in cell differentiation trajectories and transcriptomic characteristics of the resulting iMac populations, including the differences in the expression of genes implicated in inflammatory response, antigen presentation and lipid homeostasis. The results are meaningful for a better understanding of iMac differentiation processes and the proper choice of an iMac differentiation protocol for different iMac applications.

## 2. Results

### 2.1. iMac Differentiation Using EBS and 2DF Protocols: The 2DF Protocol Is More Reliable

To address the question of how the type of differentiation protocol affects iMac characteristics, we differentiated iMacs using two well established and significantly different protocols, i.e., EBS [32] and 2DF [29]. We used two different iPSC lines for iMac differentiation, iMA (derived from human fibroblasts) and K7-4Lf (K7; derived from human peripheral blood cells) [33,34]. 

**Figure 1:**
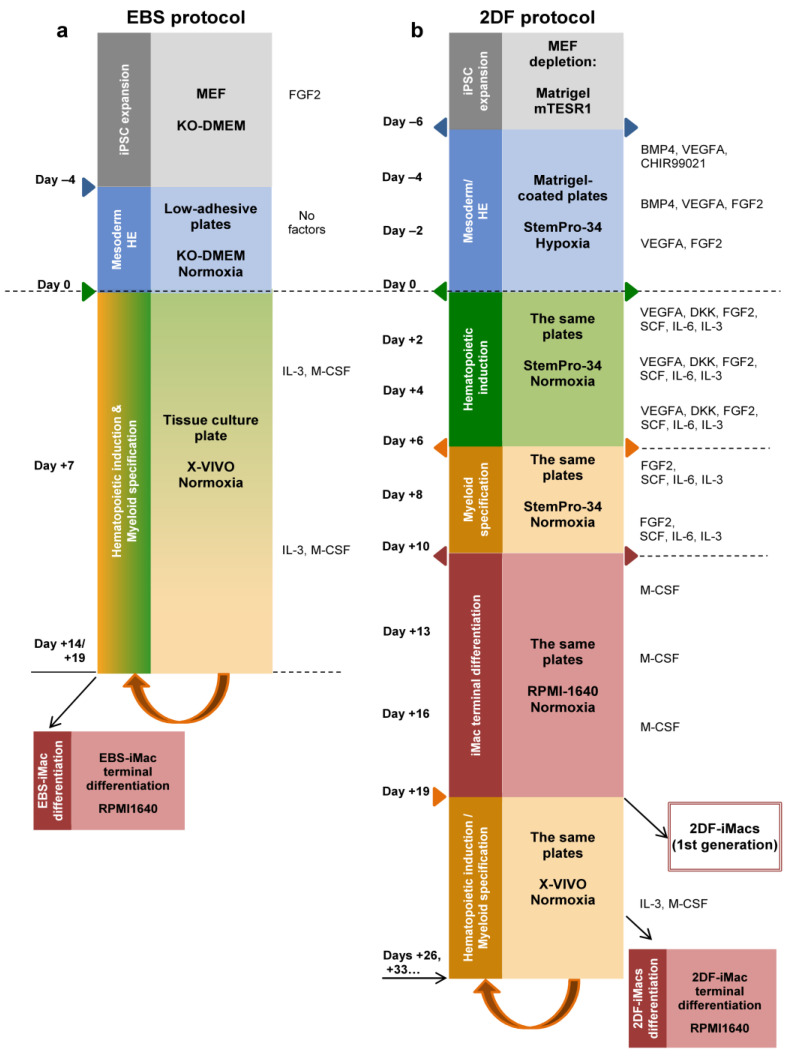
Schematic representation of iMac differentiation procedures using embryoid-body-dependent spontaneous (EBS) and embryoid-body-independent exogenous factor-dependent (2DF) differentiation protocols. (**a**) EBS protocol. induced pluripotent stem cells (iPSCs) were expanded on mouse embryonic fibroblast feeder cells (MEFs) and collected and cultured in low-adherent conditions in the absence of exogenous differentiation factors (day −4 to day 0) to induce embryoid body (EB) formation. At day 0, EBs were collected and transferred to culture tissue plates, where they were cultured in the presence of IL-3 and macrophage colony-stimulating factor (M-CSF) to induce hematopoietic specification and myeloid differentiation. When floating iMac precursors appeared in the culture (day +14 to day +19), they were collected and differentiated into EBS-iMacs in the presence of M-CSF. The remaining cultures were restimulated with IL-3/M-CSF to continue the generation of EBS-iMac precursors followed by their terminal differentiation into EBS-iMacs. (**b**) The 2DF protocol. To induce mesoderm/HE, MEF-depleted iPSCs were cultured in tissue culture plates coated with Matrigel in the presence of indicated exogenous factors (day −6 to day 0). On day 0, the composition of exogenous factors was changed in a way to induce first hematopoietic specification (day 0 to day +6) and next myeloid differentiation (day +6 to day +10). BMP4, bone morphogenetic protein 4; DKK, Dickkopf-related protein 1; FGF2, basic fibroblast growth factor; Flt3L, Fms-related receptor tyrosine kinase 3 ligand; SCF, stem cell factor; TPO, thrombopoietin; VEGFA, vascular endothelial growth factor A.

**Figure 2:**
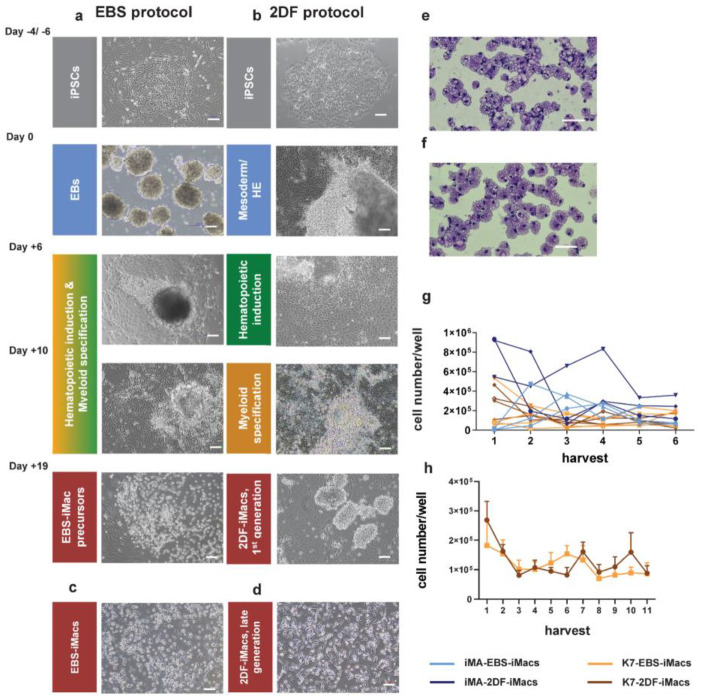
Representative morphology of differentiating cells, resulting iMac populations and harvest counts of EBS- and 2DF-iMacs. (**a**,**b**) Representative light microscopy of cell cultures at the indicated stages of iMac differentiation. (**a**) EBS protocol, (**b**) 2DF protocol. (**c**,**d**) Representative light microscopy of EBS- (**c**) and 2DF (**d**) iMacs. Phase contrast. (**e**,**f**) Giemsa staining of EBS-iMac (**e**) and 2DF-iMac (**f**) cytospins. (**a**–**f**) Scale bars represent 100 µm; shown are representative results obtained during the differentiation of iPSC line K7; similar data were obtained for iPSC line iMA. (**g**) Weekly yields of EBS- and 2DF-iMacs derived from iPSC line iMA (blue) and K7 (orange). Light colors, EBS-iMacs; dark colors, 2DF-iMacs. Most experiments were terminated at week 6. (**h**) A comparison of the duration of EBS- and 2DF-iMac generation summarized data obtained in 5 independent differentiation experiments for each type of protocol (iPSC line K7).

Briefly, in the EBS protocol, iPSCs were cultured in low-adhesive conditions to induce the formation of EBs and mesoderm (day −4 to day 0). Starting day 0, EBs were transferred to tissue culture plates and cultured in the presence of IL-3 and M-CSF (Figure 1a). Starting around day +14, monocytic iMac precursors appeared in the cultures as large floating pseudopodia-equipped cells (Figure 2a). They were harvested weekly and terminally differentiated into EBS-iMacs in the presence of M-CSF. 

In the 2DF protocol, feeder-iPSCs were cultured on Matrigel-coated surfaces in the presence of mixtures of factors, known to induce (i) mesoderm and HE (day −6 to day 0); (ii) hematopoietic commitment (day 0 to day +6); (iii) myeloid specification (day +6 to day +10); and (iv) iMac formation (starting day +10; Figure 1b). Terminally differentiated 2DF-iMacs appeared in the cultures around day +19 (Figure 2b). After their first harvest, the cultures were restimulated with IL-3 and M-CSF. This resulted in the continuous generation of 2DF-iMac precursors that were harvested weekly and terminally differentiated into 2DF-iMacs, as was performed in the EBS protocol. 

The differentiated EBS-iMacs and 2DF-iMacs had similar morphology, i.e., they were large (approximately 20 µm in diameter), vacuolated and equipped with pseudopodia (Figure 2c–f).

Our analysis of the productivity of EBS and 2DF protocols showed that 2DF differentiation experiments were always productive, whereas in 4 out of 15 EBS differentiations EBS-iMacs were generated late, in low quantities and not in all wells (the experiments were excluded from the subsequent analyses). To compare the yield efficacy of EBS and 2DF protocols, we determined the number of iMacs that could be harvested weekly from one differentiation well of a 6-well plate in a successful differentiation experiment. 

For iMA-derived iMacs, the productivity of the first two iMac harvests was higher when the cells were differentiated using the 2DF protocol (*p* = 0.029 for EBS and 2DF protocol comparison; Figure 2g). For K7-derived iMacs, no significant differences in the first iMac yields obtained in EBS and 2DF protocols were registered (*p* = 0.343). The exact reasons why the early yields of iMA- and K7-derived iMacs differentially depended on the type of protocol are not clear. Supposedly, they are due to the differences between the background iPSCs that originated from different donors and from different source cells (fibroblasts and mononuclear blood cells, respectively), and thus could differentially depend on factors driving cell differentiation. The explanation is indirectly supported by published data showing significant differences in monocyte productivity between different iPSC lines [35]. The longevity of iMac generation was comparable between the protocols (Figure 2h). 

Overall, both protocols allowed to generate iMacs, however, the 2DF protocol gave more stable results in terms of the success of iMac generation.

### 2.2. EBS-iMacs and 2DF-iMacs Display Similar Phenotype and Phagocytic Activity

**Figure 3:**
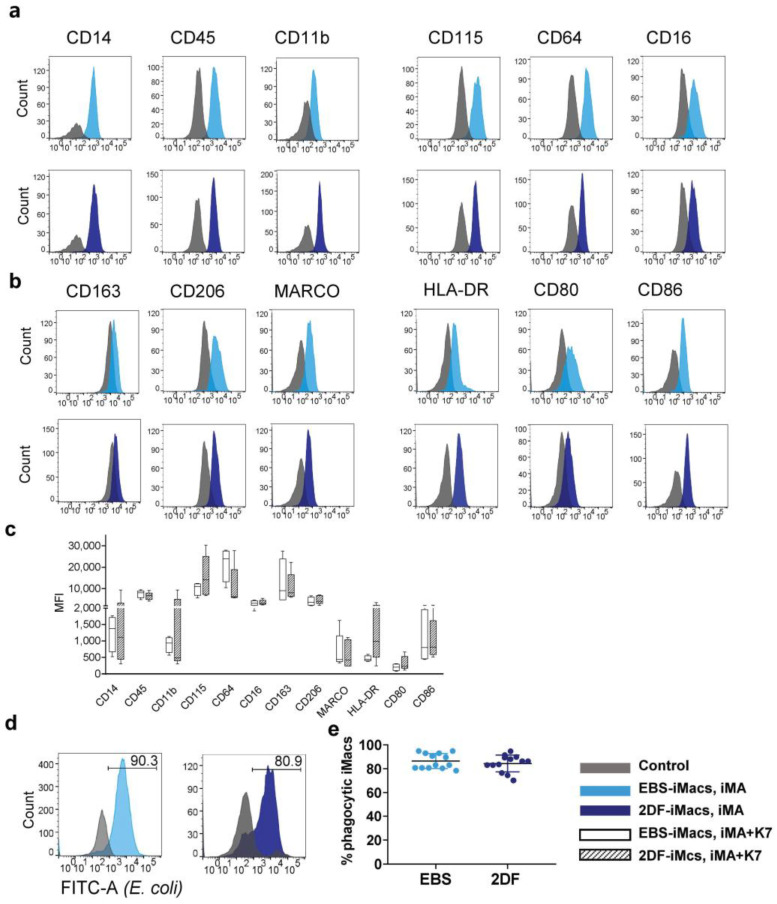
EBS- and 2DF-iMacs display similar phenotype and phagocytic activity. (**a**–**d**) EBS- and 2DF-iMacs were collected at different differentiation time points and stained with antibodies or analyzed in Phagotest^TM^ (**d**,**e**). (**a**) iMac expression of markers specific for macrophages/myeloid cells. (**b**) iMac expression of markers associated with macrophage polarization and activation. Shown are the results of one experiment obtained in iMA-derived EBS-iMacs and 2DF-iMacs. The results are representative of 7 independent experiments performed in iMA-derived (n = 3) and K7-derived (n = 4) EBS- and 2DF-iMacs; EBS-iMacs and 2DF-iMacs were differentiated and analyzed simultaneously; in each experiment, 3000 to 10,000 CD14^+^ cells were collected; for histograms, data were downsampled to 3000 events. (**c**) Mean fluorescence intensity (MFI) of the expression of analyzed markers on iMA- and K7-derived EBS- and 2DF-iMacs: summarized data obtained in 6 independent experiments (iMA, n = 3; K7, n = 3). (**d**) The comparison of the phagocytic activity of EBS- and 2DF-iMacs in Phagotest^TM^: representative histograms. (**e**) The comparison of the phagocytic activity of EBS- and 2DF-iMacs in Phagotest^TM^: summarized data (13 independent experiments; shown are percentages of phagocytic cells after gating on CD14^+^ cells, mean ± SD). Analyses were performed using iMac harvests 2–17 and gave similar results. In each experiment, at least 5000 CD14^+^ cells were collected and analyzed.

Flow cytometry analysis of terminally differentiated iMacs showed that 80–98% of the collected cells were CD14^+^. Fine phenotypic analysis demonstrated that all iMacs (i.e., iMA- and K7-derived EBS-iMacs and 2DF-iMacs) expressed a similar CD14^+^CD45^+^CD11b^+^CD115^+^CD64^+^CD16^+/low^CD163^+/low^CD206^+/low^MARCO^+/low^HLA-DR^+/low^CD86^+/low^CD80^low^ phenotype (Figure 3a–c) that can be interpreted as a phenotype of non-polarized “naïve-like” macrophages [32]. 

iMac phagocytic capacity was evaluated using Phagotest^TM^. Both EBS-iMacs and 2DF-iMacs displayed similarly high phagocytic activity, which was preserved at late iMac harvests (Figure 3d,e). 

### 2.3. iMac Secretory Profile Is Characterized by the Co-Production of Pro- and Anti-Inflammatory Factors and Depends on the Differentiation Protocol and Source iPSC Line 

**Figure 4:**
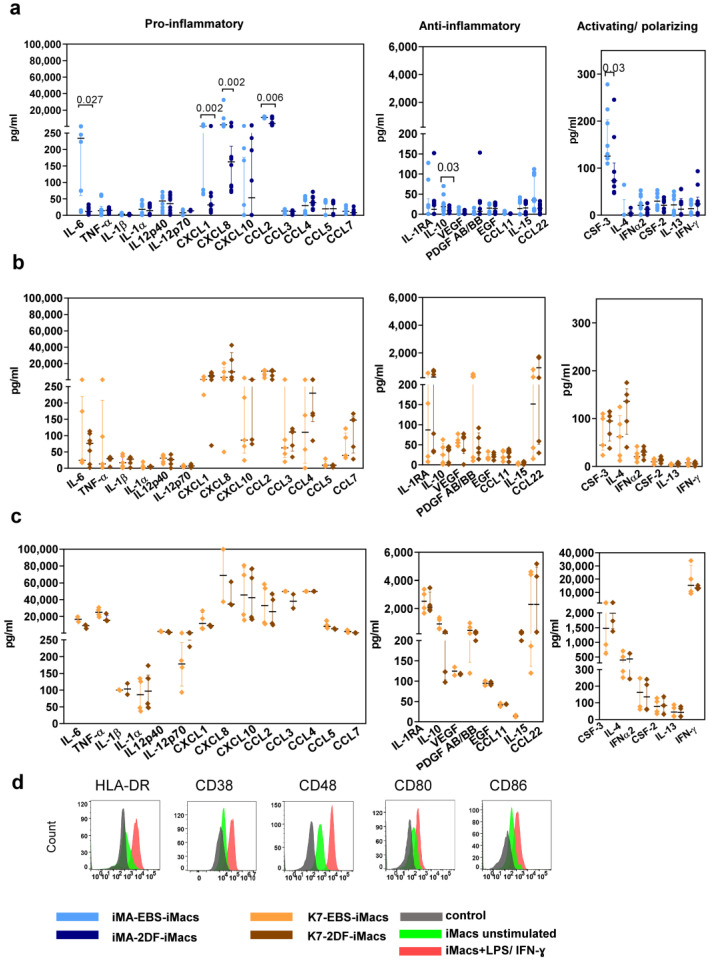
EBS- and 2DF-iMacs are responsive to inflammatory stimuli. EBS- and 2DF-iMacs were left unstimulated or were stimulated with LPS (100 ng/mL) and IFN-γ (20 ng/mL); 24 h later, the supernatants were collected. The cells were stained with antibodies specific to HLA-DR, CD38, CD48, CD80 and CD86. (**a**,**b**) The baseline secretion of indicated cytokines by EBS- and 2DF-iMacs (medians and interquartile ranges). (**a**) iMA-derived iMacs; (**b**) K7-derived iMacs. (**c**) The secretion of indicated cytokines by K7-derived iMacs stimulated with LPS/IFN-γ. Similar results were obtained using iMA-derived iMacs. The supernatants were collected from iMacs generated in 3 independent iMA and 3 independent K7 differentiation experiments. In each differentiation experiment, the supernatants were collected from 1–2 iMac harvests; all supernatants were analyzed simultaneously. (**d**) Surface expression of HLA-DR, CD38, CD48, CD80 and CD86 by EBS- and 2DF-iMacs (representative data of 3 independent experiments obtained in K7-derived iMacs).

iMac secretory profiles were evaluated by measuring the basal levels of 41 cytokines in the supernatants of iMA- and K7-derived EBS-iMacs and 2DF-iMacs. All iMacs co-produced pro- and anti-inflammatory cytokines, supporting our conclusion on their unpolarized “naïve-like” state. The basal secretory profiles of iMA-derived iMacs showed inter-protocol differences: compared with 2DF-iMacs, EBS-iMacs exhibited a higher-level production of CXCL1, CXCL8, CCL2, IL-6, CSF3 and IL-10 (Figure 4a). The basal secretory profiles of K7-derived EBS-iMacs and 2DF-iMacs were similar (Figure 4b).

In response to the stimulation with LPS and IFN-γ, iMacs significantly increased the secretion of the main pro-inflammatory cytokines (e.g., IL-6, TNF-α, CCL2, *p* < 0.0001 for iMA- and *p* < 0.01 for K7-derived iMacs) and some anti-inflammatory factors (IL-10, PDGFAB/BB, *p* < 0.0001 for iMA- and *p* < 0.02 for K7-derived iMacs; Figure 4c). The responsiveness of iMacs to LPS/IFN-γ was also confirmed by a significant up-regulation of the surface expression of HLA-DR, CD38, CD48, CD80 and CD86 (Figure 4d). 

Overall, EBS- and 2DF-iMacs derived from different iPSC lines displayed a similar phenotype of non-polarized macrophages, were highly phagocytic and responsive to inflammatory stimuli. At the same time, the basal cytokine profile of iMacs depended on the source iPSC line and the type of differentiation protocol.

### 2.4. The Transcriptomic Profiles of EBS- and 2DF-iMacs Differ by the Expression of Genes Involved in Immune Response, Antigen Presentation and Lipid Homeostasis

To obtain a deeper insight into the identity of EBS-iMacs and 2DF-iMacs, we compared their transcriptomic profiles. EBS-iMacs and 2DF-iMacs were differentiated in parallel, from the same source of iPSCs. At the first iMac harvest (i.e., on days 19–24), the cells were collected, FACS-sorted for CD14^+^ population and used for RNA isolation. In total, we performed four independent differentiation experiments using iPSC line iMA and three independent differentiation experiments using iPSC line K7. This resulted in the successful on-time generation of two populations of iMA-derived EBS-iMacs, four populations of iMA-derived 2DF-iMacs, two populations of K7-derived EBS-iMacs and three populations of K7-derived 2DF-iMacs (in two differentiation experiments from iMA-iPSCs and one differentiation experiment from K7-iPSCs, EBS-iMacs formed late, and they were not included in the analysis). In total, 11 cDNA libraries were established. For each library, 34.0M to 48.8M reads were produced; low-quality reads were removed, which resulted in 33.9 to 48.7 reads (99.6–99.9%). In total, 25.9M to 36.9M (75.9–76.3%) reads were uniquely mapped on the reference genome. The experiment-related batch effect was modeled within the DESeq2 study design [36]; the batch effect from log-normalized data was removed using the removeBatchEffect function from limma [37].

**Figure 5:**
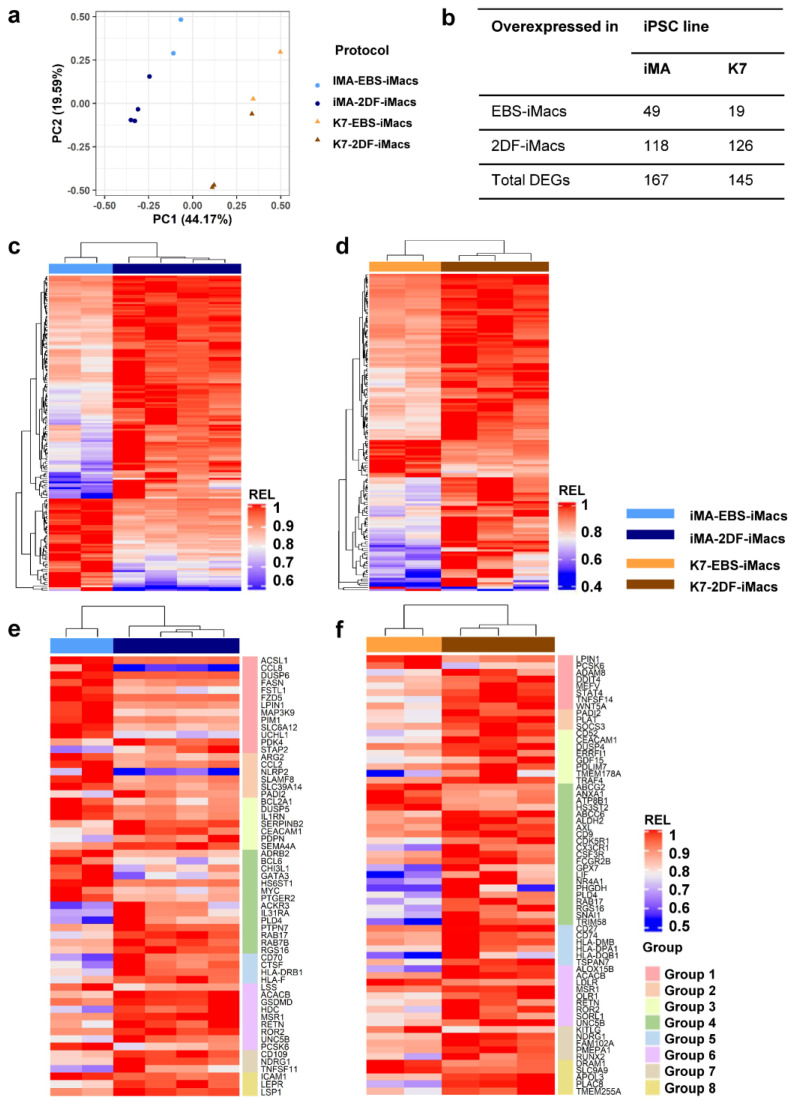
EBS- and 2DF-iMacs differ by fine transcriptomic characteristics. EBS- and 2DF-iMacs were differentiated in parallel in 4 (iMA) and 3 (K7) independent differentiation experiments. RNA was isolated from the resulting CD14+ sorted iMacs (provided they were formed on time) and sequenced (two batches of iMA-EBS-iMacs and one batch of K7-EBS-iMacs were generated late and were excluded from the analysis). (**a**) Principal component (PC) analysis showing the separation of iMacs based on the source iPSC line (PC1) and the type of differentiation protocol (PC2). (**b**) Numbers of differentially expressed genes (DEGs) between iMA- and K7-derived EBS-iMacs and 2DF-iMacs. (**c**,**d**) Heatmaps depicting the expression of DEGs in iMA- (**c**) and K7- (**d**) derived EBS-iMacs and 2DF-iMacs. Expression levels were normalized on the maximal value separately for (i) each gene and (ii) EBS and 2DF differentiation experiments. (**e**,**f**) Heatmaps depicting the expression of selected DEGs between EBS- and 2DF-iMacs. (**e**) iMA-derived iMacs; (**f**) K7-derived iMacs. DEGs were categorized into 8 functional groups based on gene role in macrophage functionality taken from available published sources. Group 1, genes induced by M1/inflammatory stimuli and involved in pro-inflammatory response; group 2, genes induced by M1/inflammatory stimuli for which both pro- and anti-inflammatory effects have been reported; group 3, genes induced by M1/inflammatory stimuli and involved in the negative regulation of inflammation; group 4, genes associated with M2/TAM macrophages and anti-inflammatory activity; group 5, genes implicated in antigen presentation, endosome functioning and costimulation; group 6, genes involved in lipid homeostasis and foam macrophage formation; group 7, genes implicated in osteoclastogenesis; group 8, genes implicated in phagocytosis and antibacterial response. For detailed description of genes, see Tables S2.1–S2.3.

A principal component (PC) analysis showed that the first PC explained the differences between the parent iPSC lines (PC1, 44.2% variability), while the second PC split the samples based on the differentiation protocol (PC2, 19.6% variability; Figure 5a). It has previously been noted that the characteristics of differentiated cells may depend on the source iPSC line [38]. In contrast, the influence of the differentiation protocol on iMac properties has not been evaluated. Therefore, we focused on the transcriptomic comparison of EBS- and 2DF-iMacs. 

iMA-derived EBS-iMacs and 2DF-iMacs differed by the expression of 167 genes, of which 49 were overexpressed in EBS-iMacs and 118 were overexpressed in 2DF-iMacs (Figure 5b–d). Genes overexpressed in iMA-EBS-iMacs were mainly associated with inflammatory responses (categories from Gene Ontology (GO) and other databases: “cellular response to interleukin-1”, “cellular response to tumor necrosis factor” and “inflammatory response”, Table 1 and Table S1). Because the same gene/pathway may play different roles in different types of cells, we searched for available data on the role which each individual overexpressed gene plays specifically in macrophages. The results allowed us to segregate differentially expressed genes (DEGs) into several functional groups, i.e., (i) genes induced by M1/inflammatory stimuli and involved in pro-inflammatory response (*ACSL1, CCL8, DUSP6, FASN, FSTL1, FZD5, LPIN1, MAP3K9, PIM1, SLC6A12* and *UCHL1;* Figure 5e, group 1; Tables S2.1–S2.3); (ii) genes induced by M1/inflammatory stimuli for which both pro- and anti-inflammatory effects have been reported (*ARG2, CCL2, NLRP2, SLAMF8* and *SLC39A14*; group 2); (iii) genes induced by M1/inflammatory stimuli and involved in the negative inflammation control (*BCL2A1*, *DUSP5* and *IL1RN;* group 3); and (iv) genes associated with M2/TAM macrophages and anti-inflammatory activity (*ADRB2, BCL6, CHI3L1, GATA3, HS6ST1, MYC* and *PTGER2*; group 4). A few genes overexpressed in iMA-EBS-iMacs were associated with lipid homeostasis (*ACSL, FASN, LPIN1* and *LSS*; group 6, some of these genes were also included in group 1), macrophage phagocytosis and antibacterial response (*CHI3L1, ICAM1* and *LSS*—positive regulation, *PTGER2* and *SLAMF8*—negative regulation; group 8; some gene were also included in other groups). The most represented of these groups was group 1, which included 11 genes with pro-inflammatory activity. The gene composition for each group and a brief description of each gene are presented in Tables S2.1–S2.3). 

Genes overexpressed in iMA-derived 2DF-iMacs belonged to multiple different categories associated with C2H2 zinc finger proteins (i.e., “ZN_FING:C2H2-type 10”, “Herpes simplex virus 1 infection”, etc.; Table 1 and Table S1). Individual gene analysis showed that compared with iMA-EBS-iMacs, in iMA-2DF-iMacs, significantly fewer genes were associated with pro-inflammatory activity (*MSR1, PDK4* and *STAP2*). Instead, overexpressed genes were related to the following: (i) M2/TAM macrophages and anti-inflammatory activity (*ACKR3, IL31RA, PLD4, PTPN7, RAB17, RAB7B, RGS16* and *HLA-F;* Figure 5e, group 4; Tables S2.1–S2.3); (ii) antigen presentation, endocytic compartment and costimulation (*CD70, CTSF, HLA-DRB1, HLA-F* and *RAB7B*; group 5) and (iii) lipid homeostasis (*ACACB, GSDMD, HDC, MSR1, RETN, ROR2, UNC5B* and *PCSK6*; group 6). Of note, all genes from the latter group, except for *ACACB*, are directly involved in macrophage uptake of low-density lipoproteins (LDL) and/or foam macrophage formation and atherosclerosis. Several genes were associated with osteoclast differentiation (*CD109, NDRG1, RETN* and *TNFSF11*; group 7, *RETN* was also included in group 6), phagocytosis and anti-bacterial activity (*LEPR, LSP1, MSR1* and *RETN;* group 8, some genes were also included in other groups; for more information, see Tables S2.1–S2.3). 

K7-derived EBS-iMacs and 2DF-iMacs differed by the expression of 145 genes. Of them, 19 genes were overexpressed in EBS-iMacs and 126 genes were overexpressed in 2DF-iMacs. Genes overexpressed in K7-EBS-iMacs did not form any significant GO term. Genes overexpressed in K7-2DF-iMacs were enriched for GO terms/other categories related to cell endosome compartment and antigen presentation (“endocytic vesicle membrane”, “MHC class II protein complex”, etc.; Table 1 and Table S1). Individual gene analysis showed that in addition to genes involved in antigen presentation and costimulation (*HLA-DPA1, HLA-DQB1, HLA-DMB, CD74* and *CD27*), in K7-2DF-iMacs there was a clear predominance of genes implicated in the following: (i) the formation of M2/TAM macrophages and anti-inflammatory activity (*AXL, FCGR2B, LIF, PLD4*, etc.; Figure 5f, group 4) and (ii) lipid homeostasis (group 6). The latter included genes involved in LDL uptake (*LDLR, MSR1, OLR1, RETN, ROR2* and *SORL1*) and foam macrophage formation (*ALOX15B* and *UNC5B*). Only a few group 6 genes exert an anti-atherogenic effect (*ABCC6, ALDH2* and *NR4A1*). Several overexpressed genes were associated with the differentiation of osteoclasts (*NDRG1, FAM102A, PMEPA1, RETN, RUNX2* and *TMEM178A*; group 7), phagocytosis and antibacterial response (*APOL3, FCGR2B, MSR1, PLAC8, RETN, STAT4* and *TMEM255A*; group 8; for more detailed information on each gene, see Tables S2.1–S2.3).

In summary, iMA-derived EBS-iMacs displayed a higher inflammatory state compared with iMA-2DF-iMacs. The 2DF-iMacs derived from both iPSC lines were characterized by an overexpression of genes associated with M2/TAM macrophages and negative inflammation control, antigen presentation and lipid homeostasis.

### 2.5. Dynamic Transcriptomic Profiles of cells Differentiating Using EBS and 2DF Protocols Differ by the Expression of Genes Involved in the Inflammatory Response and Lipid Homeostasis 

To find out whether the differentiation of iMacs goes through similar or different trajectories in EBS and 2DF protocols, we next performed dynamic RNA sequencing of differentiating cells. iMacs were differentiated from iPSC lines iMA (two independent EBS/2DF differentiation experiments) and K7 (one EBS/2DF differentiation experiment); RNA was isolated on days −4/−6, 0, +6, +10 and +19 of iMac differentiation (hereafter marked as d_−4_, d_−6_, d_0_, etc.) To focus on iMac source cells in our analysis and to exclude maturing iMacs/iMac precursors from the examination, before isolating RNA, we removed all floating cells from the cultures and isolated RNA from adherent cells only. 

To analyze RNA-seq results, we used three different approaches. First, we analyzed how the expression of genes changed within each protocol between each two adjacent time points, i.e., between days −4/−6 (iPSCs) and day 0 (iPSCs/d_0_), as well as day 0 and day +6 (d_0_/d_+6_), etc. The results of all three differentiation experiments were included in the analysis. Genes whose expression changed significantly at each differentiation stage within a given protocol (i.e., EBS or 2DF) were determined using DESeq2 and referred to as “within-protocol pairwise DEGs”. Second, we directly searched for genes whose dynamic expression differed between the protocols. For that, RNA-seq data obtained in all three differentiation experiments in both protocols at all differentiation time points were included in the analysis and DEGs were identified using DESeq2 (“inter-protocol DEGs”). Finally, we included in the analysis the same RNA-seq data as in the second approach and performed pathway analysis investigating gene sets of the human Hallmark collection from MSigDB. The gene set co-regulation analysis (GESECA) allowed us to assess the contribution of each pathway to the expression variability of the data (“inter-protocol pathways”).

**Figure 6:**
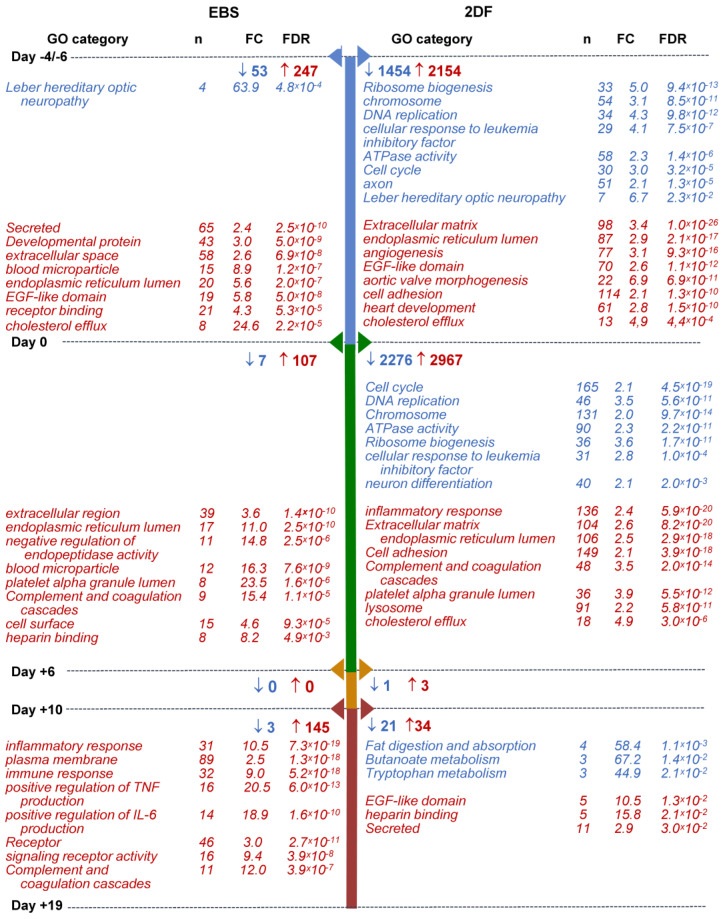
Selected categories of genes down- and up-regulated following iPSC to iMac differentiation using EBS (left) and 2DF (right) protocols (intra-protocol comparison). Three parallel EBS and 2DF differentiation experiments were performed using iMA (n = 2) and K7 (n = 1) iPSCs. RNA and 2DF differentiation experiments were performed using iMA (n = 2) and K7 (n = 1) iPSCs. RNA was isolated from iPSCs and differentiating cells on days d_−4/−6_, d_0_, d_+6_, d_+10_ and d_+19_; genes that significantly changed their expression in all three differentiation experiments at each differentiation stage within a given protocol (“intra-protocol pairwise DEGs”) were determined using DESeq2. GO terms and other categories shown in the figure were selected based on the following criteria: (i) are referred to in Gene Ontology (GO), Kyoto Encyclopedia of Genes and Genomes (KEGG) or UniProtKB Keywords (UP-KW); (ii) the false discovery rate (FDR) is <0.05; (iii) fold change (FC) is >2; (iv) if several structurally or functionally similar GO terms/other categories met criteria i-iii, only one of them (having the lowest FDR) was selected. Blue, GOs and other categories down-regulated at the indicated differentiation stages; red, GOs and other categories up-regulated at the indicated differentiation stages. For the entire list of GO terms and other categories, see Tables S4.1–S4.4.

Within-protocol pairwise gene analysis showed the following: (i) that the most numerous changes in gene expression occurred at the early (iPSCs/d_0_ and d_0_/d_+6_) differentiation stages; (ii) that in both protocols and at all differentiation stages, more genes were up-regulated than down-regulated; and (iii) that the 2DF protocol induced more pronounced alterations in gene expression compared with the EBS protocol. Indeed, during EBS differentiation, the numbers of down-regulated DEGs were 53, 7, 0 and 3 for day-pairs iPSCs/d_0_, d_0_/d_+6_, d_+6_/d_+10_ and d_+10_/d_+19_, respectively; the numbers of up-regulated DEGs were 247, 107, 0 and 145, respectively. During 2DF differentiation, the corresponding numbers of DEGs were 1454, 2276, 1 and 21 for down-regulated and 2154, 2967, 3 and 34 for up-regulated genes (Figure 6; Tables S3.1 and S3.2). 

GO terms/other categories significantly down-regulated at the early (iPSCs/d_0_ and d_0_/d_+6_) stages of 2DF differentiation were mainly related to DNA replication (“DNA replication”, “CMG complex”, etc.), ribosome functioning (“Ribosome biogenesis”, “rRNA processing”, etc.), cell division (“Cell cycle”, “mitotic cell cycle phase transition”, etc.) and cell energy supply (“ATPase activity”; Figure 6, Tables S4.1 and S4.2). The list of down-regulated genes included *ORC1, MSM2, CDC45* and *DBF4* (responsible for the initiation of DNA replication), *TTK* (chromosome alignment at the centromere), *CCNA2* (G1/S and the G2/M transition), *BUB1, BUB3* (spindle assembly), *PTTG1* (chromosome separation), etc. Alteration in their expression likely mirrored a decline in cell pluripotency. Additionally, the expression of genes associated with neuronal differentiation also contracted at the early differentiation stages (“axon”, “chemical synaptic transmission”, “Postsynaptic cell membrane”, etc.), which we interpreted as a sign of a decline in cell ectoderm differentiation potential. In contrast to 2DF differentiation, at the early stages of EBS differentiation, there were no significantly down-regulated GO terms/other categories except for “Leber hereditary optic neuropathy”. This was likely related to the fact that in the EBS protocol, biological changes were more extended in time, as a result of which time-pairwise comparison did not pass the the false discovery rate (FDR) < 0.05 threshold. 

Among GO terms/other categories up-regulated at the early differentiation stages, several categories were common for both protocols. These were related to endoplasmic reticulum, extracellular region, cell receptors and cell adhesion, which likely marked a start of active cell differentiation; other common changes included the expression of genes linked to platelets and coagulation (e.g., “platelet alpha granule lumen”, “Complement and coagulation cascades” and “Blood coagulation”) and lipid homeostasis (“positive regulation of cholesterol esterification”, “cholesterol efflux”, etc., Figure 6, Tables S4.1 and S4.2). Protocol-specific early (iPSCs/d_0_) expression changes included an up-regulation of genes belonging to multiple different developmental processes during the iPSCs/d_0_ differentiation stage. The related categories were much more numerous in 2DF compared with EBS differentiation and included “aortic valve morphogenesis”, “heart development”, “skeletal system development”, “ventricular septum morphogenesis”, “vasculogenesis”, “lung development” and other categories (Table S4.1). Of note, at d_+6_ these categories were no longer significantly enriched, pointing to a narrowing of cell differentiation potential at such an early differentiation stage as between days d_0_/d_+6_.

Along with this (d_0_/d+_6_), multiple different categories associated with inflammatory response, innate immunity and immune cell functioning up-regulated during 2DF differentiation (“Innate immunity”, “inflammatory response”, “cellular response to lipopolysaccharide”, etc.; Figure 6, Table S4.2). During EBS differentiation, the overexpression of immune-response-associated categories was noticed much later, i.e., at the stage d_+10_/d_+19_ (see Tables S4.1–S4.4 for the entire list of categories enriched at each differentiation stage).

**Figure 7:**
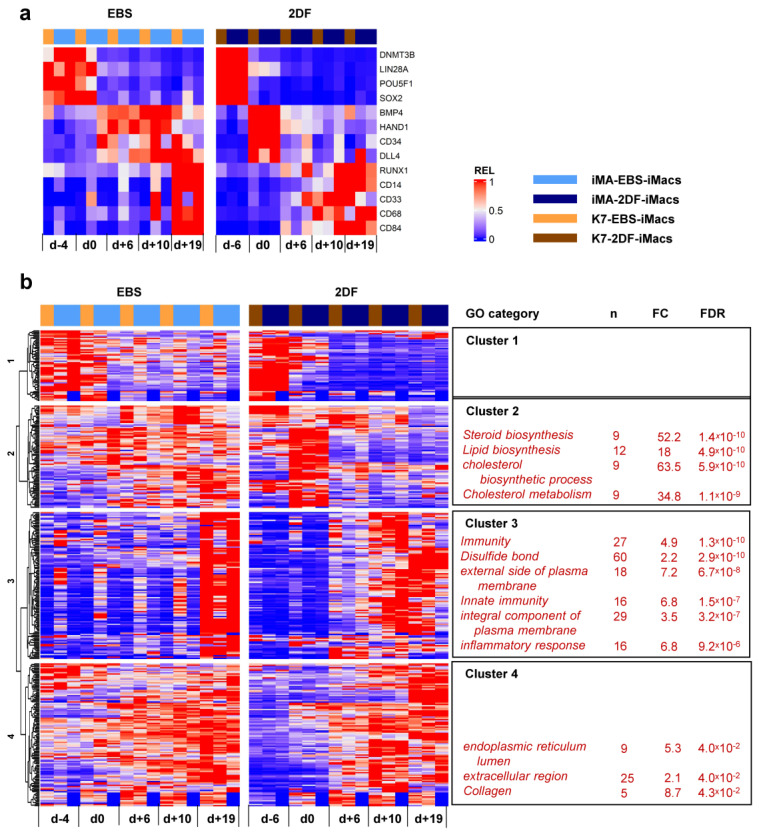
Dynamic transcriptomic profiles of cells undergoing differentiation using EBS and 2DF protocols (inter-protocol differences). The cells were differentiated, and RNA was isolated as described in Figure 6. (**a**) Dynamic changes in the expression of selected genes associated with (i) cell pluripotency (*DNMT3B, LIN28A, POU5F1* and *SOX2*); (ii) mesoderm (*BMP4* and *HAND1*); (iii) hemogenic endothelium and hematopoietic precursors (*CD34, DLL4* and *RUNX1*); and (iv) myeloid cells/macrophages (*CD14, CD33, CD68* and *CD84*). (**b**) Inter-protocol DEGs identified by applying DESeq2 to all RNA-seq data obtained in three differentiation experiments in both protocols at all differentiation time points. For each cluster, selected GO terms are listed.

To look purposefully at stage-specific changes during EBS and 2DF differentiation, we made a heatmap showing the dynamic expression of selected individual genes associated with (i) cell pluripotency (*DNMT3B, LIN28A, POU5F1* and *SOX2*); (ii) mesoderm (*BMP4* and *HAND1*); (iii) hemogenic endothelium and hematopoietic precursors (*CD34, DLL4* and *RUNX1*); and (iv) myeloid cells/macrophages (*CD14, CD33, CD68* and *CD84*). Compared with EBS differentiation, 2DF differentiation induced a more rapid decline in the expression of pluripotency and mesoderm-associated genes and an earlier up-regulation of myeloid cell markers (Figure 7a).

Inter-protocol DESeq2 analyses identified 795 DEGs, of which 366 genes had fold change (FC) > 2. Unsupervised hierarchical clustering analysis identified four main DEG clusters (Figure 7b). 

Cluster 1 contained 56 genes whose expression declined during the differentiation process. During 2DF differentiation, a decline occurred earlier compared with EBS differentiation. The cluster included a number of genes implicated in various different aspects of cell differentiation and functioning, such as neurogenesis (*CHAC1, GPC2, GDAP1L1, INA* and *NETO1*), cytoskeleton organization (*FSD1, STRIP2* and *SEPT3*), lipid metabolism (*FASN* and *PNPLA3*), etc. However, the genes of this cluster did not form any significantly enriched GO term.

Cluster 2 contained 79 genes whose expression in 2DF differentiation experiments transiently increased by d_0_ and declined afterwards; in EBS experiments, the expression of cluster 2 genes was more miscellaneous and lasted longer. Most significantly enriched GO categories were associated with lipid/cholesterol biosynthesis and included such genes as *HMGCR, HMGCS1, LSS, MSMO1, MVD* and *MVK*.

Cluster 3 contained 113 genes which were initially expressed at low levels and were significantly up-regulated during the differentiation process. In 2DF experiments, the expression of Cluster 3 genes increased by d_+6_ or /d_+10_. In EBS experiments, expression profiles changed less synchronously and reached maximal levels only by d_+19_. The main significantly enriched GOs were related to various aspects of inflammatory response and immune defense (“immunity”, “innate immunity”, “inflammatory response”, “Phagosome”, etc.) as well as to plasma membrane receptor apparatus (“external side of plasma membrane”, “integral component of plasma membrane”, etc.). The corresponding genes included B2M, CD14, CD33, CD48, CD86, CSF2RB, CSF3R, FCER1G, FCGR2B, *TLR2*, etc. (for the whole list of significantly enriched GOs and corresponding DEGs in each cluster, see Tables S5.1 and S5.2).

Finally, Cluster 4 contained 116 genes whose expression also increased following the differentiation, but at earlier stages compared with Cluster 3 genes. The genes included *COL4A1, COL4A2, CD55*, Serpins and other genes related to collagen, the extracellular region and endoplasmic reticulum lumen.

**Figure 8:**
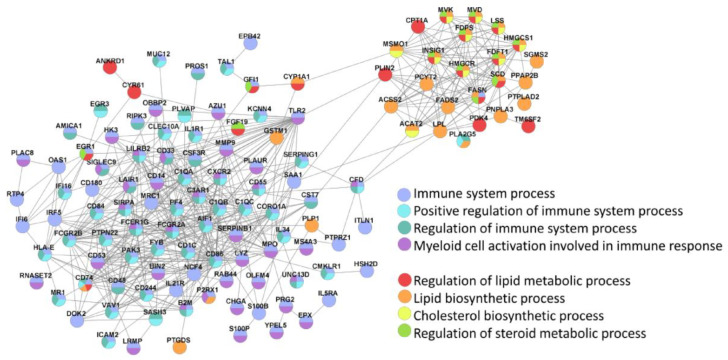
STRING identifies two main clusters of inter-protocol differentially expressed genes that are associated with immune response and lipid homeostasis. All inter-protocol DEGs (Figure 7b) were uploaded to STRING. The input options included a confidence cutoff of 0.4 and maximum additional interactions of 0. The GO Biological Processes with FDR < 4.0 × 10^−6^ are shown.

The main two clusters of inter-protocol DEGs, one associated with immunity and the other related to lipid homeostasis, were also identified in the Search Tool for Retrieval of INteracting Genes (STRING; (Figure 8).

**Figure 9:**
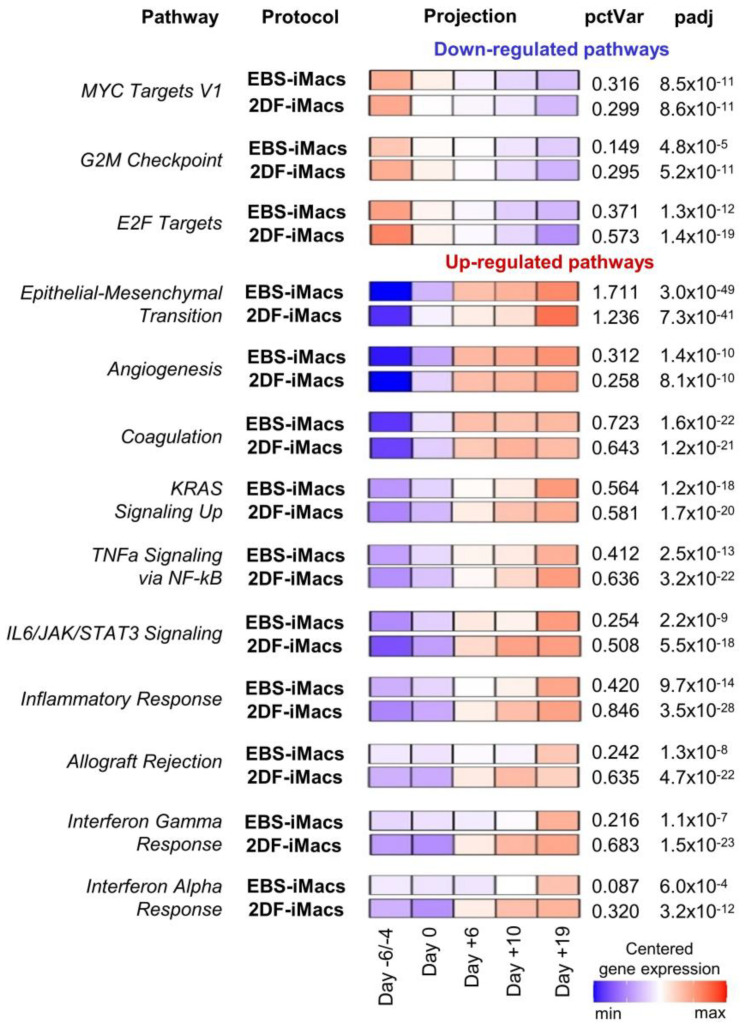
Pathway-level analysis highlights dynamic expression changes in cells differentiating in EBS and 2DF protocols. RNA-seq data obtained in two independent differentiation experiments were analyzed using the gene set co-regulation analysis (GESECA) which computes percent of explained variance for each of the gene sets (pathways) from human Hallmark collection of Molecular Signatures Database (each differentiation experiment included parallel differentiation of iPSC line iMA using EBS and 2DF protocols). Significant pathways associated with cell proliferative and immunological activity and differentiation processes are shown (inter-protocol pathways). Projection illustrates centered gene expression dynamics with colors scaled for each protocol individually from minimal to maximal value of the analyzed expression matrix; pctVar—percent of variance in the data that the corresponding pathway explains; padj—adjusted *p*-value. Similar results were obtained for the differentiation of iPSC line K7 (one differentiation experiment, the results are presented in Figure S1).

Inter-protocol pathway analysis of the differentiation of iPSC line iMA revealed 26 pathways with significant variations during the differentiation process (Figure 9; Tables S6.1–S6.4). Down-regulated pathways included cell-proliferation-related gene sets (i.e., “MYC Targets V1”, “G2M Checkpoint” and “E2F Targets”). These pathways exhibited similar expression dynamics in both EBS and 2DF differentiations (Figure 6).

Up-regulated pathways were related to developmental processes (e.g., “Epithelial-Mesenchymal Transition”, “Angiogenesis” and “Coagulation”), signaling pathways linked to inflammatory response and cell proliferation (e.g., “KRAS Signaling Up”, “TNFa Signaling via NF-kB” and “IL6/JAK/STAT3 Signaling”) and immunological changes in differentiating cells (e.g., “Inflammatory Response”, “Interferon Gamma Response” and “Interferon Alpha Response”). “Epithelial-Mesenchymal Transition” and “Coagulation” pathways were at the top of the list (by the percent of explained variance (Figure S1)). Of note, genes involved in the “Epithelial-Mesenchymal Transition” pathway were activated with a smoother time-dependent expression dynamic in the EBS protocol compared with a strong expression growth at the end of the differentiation in the 2DF protocol. In contrast, genes of immune-related pathways underwent an earlier up-regulation in the 2DF protocol (Figure S1).

Among pathways significantly explaining variations in differentiation experiments, pathways related to lipid homeostasis were also present (“Cholesterol homeostasis”, “Fatty acid metabolism” etc.). These pathways explained a relatively small percentage of variation and they prevailed in the K7 lineage in terms of their number. Nevertheless, inter-protocol differences in the dynamic expression of these pathways were detected: in EBS experiments, the expression increased gradually from the first to the last differentiation stage; in the 2DF differentiation, the maximal expression rise was noted in the middle of the differentiation process (Tables S6.1–S6.4, Figure S1).

The main trends in the dynamics of pathway expression seen in iMA cells were also observed in differentiating K7 cells (one differentiation experiment, the results are shown in Figure S1).

Overall, transcriptionally, iMac differentiation trajectories were characterized by the following: (i) stage-specific changes in the expression of genes related to cell pluripotency, mesoderm, hematopoietic specification and myeloid differentiation; (ii) early down-regulation of genes/pathways involved in cell division; (iii) up-regulation of immune response/inflammation-related genes/pathways. Compared with the EBS protocol, 2DF differentiation demonstrated more rapid and clearer patterns of gene expression dynamics; in particular, this applied to the expression of “immune” genes. iMac differentiation was also accompanied by significant alterations in the expression of genes involved in lipid/cholesterol homeostasis, whose dynamic expression differed between the protocols.

## 3. Discussion

In this study, we performed the first direct comparison of iMacs generated from the same iPSC lines using two different protocols, EBS and 2DF. We found that although EBS-iMacs and 2DF-iMacs display similar general macrophage-like characteristics (i.e., macrophage-like morphology, surface phenotype and high phagocytic activity), they differ by fine transcriptomic characteristics as well as by the dynamic transcriptome of their differentiating progenitors.

One of the differences between terminally differentiated EBS- and 2DF-iMacs concerned their inflammatory potential. Previous studies have convincingly demonstrated that iMacs generated using diverse protocols display the characteristics of M0 macrophages slightly biased to an M2 state [3,26,32,39]. Our data on the phenotype and the secretory profiles of EBS- and 2DF-iMacs agree with these previous reports, as we found the co-expression of M1 and M2 markers and the co-production of pro- and anti-inflammatory cytokines by EBS- and 2DF-iMacs. At the same time, in our study, iMA-derived EBS-iMacs displayed a higher-level secretion of several pro-inflammatory cytokines (i.e., CCL2, IL-6, CXCL1 and CXCL8) compared with iMA-derived 2DF-iMacs. At mRNA level, iMA-EBS-iMacs overexpressed a set of genes involved in the positive regulation of inflammation (e.g., *CCL2, CCL8, FASN* and *LPIN1*), and these iMacs were significantly enriched for inflammation-related categories. In turn, 2DF-iMacs were enriched for genes associated with M2 macrophages and the negative regulation of inflammation. Thus, we concluded that EBS-iMacs were prone to a higher inflammatory activity, whereas 2DF-iMacs were more skewed toward an anti-inflammatory M2-biased state.

Another difference between EBS- and 2DF-iMacs concerned the expression of genes associated with antigen presentation. These were stably over-expressed in 2DF-iMacs and included genes directly implicated in the presentation of exogenous antigens (*HLA-DRB1, HLA-DPA1* and *HLA-DQB1*) as well as genes performing accessory functions, such as the stabilization of peptide-free MHC class II (*CD74*), the degradation of MHC class II-associated invariant chain to CLIP (*CTSF),* the loading of exogenous peptides into MHC class II cleft (*HLA-DMB*) and the formation of membrane tetrasponin-enriched microdomains and endocytosis (*TSPAN7* and *TSPAN16*) (Tables S2.2 and S2.3 for the corresponding references). Previously, several groups showed a low-level expression of MHC class II molecules by iMacs [3,22,32,40]. It was also demonstrated that in response to LPS/IFN-γ, iMacs increase MHC class II expression, indicating that they are capable of effective antigen presentation under appropriate conditions ([41], similar results were also obtained in the present study). Our study extends these observations by demonstrating for the first time that iMac basal antigen presentation potential may depend on the type of protocol used for their differentiation. 

Finally, the transcriptomic profile of EBS- and 2DF-iMacs differed by the expression of genes implicated in lipid homeostasis. EBS-iMacs overexpressed only a few genes of this group, i.e., *FASN* (catalyzes de novo synthesis of long-chain saturated fatty acids), *LSS* (catalyzes the conversion of (S)-2,3 oxidosqualene to lanosterol), *LPIN1* (converts phosphatidate to diacylglycerol), *PSCK6* (inactivates lipoprotein lipase) and *ACSL1* (Acyl-CoA synthetase-1). In contrast, in 2DF-iMacs, notably more genes related to lipid homeostasis were overexpressed. Most of them are known as being involved in LDL/oxidized LDL uptake (*LDLR, MSR1, OLR1, RETN, ROR2* and *SORL1*) and foam macrophage formation (*ALOX15B, GSDMD, HDC* and *UNC5B*, etc.), and only three genes possess an anti-atherogenic effect (*ABCC6, ALDH2* and *NT4A1*). Thus, 2DF-iMacs were transcriptionally predisposed to LDL/cholesterol accumulation and the conversion to foam macrophages. 

Lipid storage, foam macrophage formation and inflammation represent key and inter-related pathogenic factors of atherosclerosis [42,43,44]. In this regard, our data on a bias of 2DF-iMacs towards both lipid accumulation and anti-inflammatory activity seem to be mismatched. However, recent findings indicate that (i) pro-inflammatory signals involved in atherosclerotic plaque formation are extrinsic, derived from the artery wall rather than from macrophages and (ii) cholesterol and lipid drop accumulation endows macrophages with anti-inflammatory/wound healing properties. Indeed, the formation of foam macrophages was shown to suppress the expression of inflammatory genes in the cells [45]; lanosterol accumulation in macrophages decreased IFNβ-mediated STAT1-STAT2 activation, inflammatory cytokine secretion and mortality from endotoxemic shock [46]; cholesterol loading of M2-polarized macrophages suppressed their pro-inflammatory activity [47]; and cholesterol and fatty acid biosynthetic processes were associated with a high speed of wound healing and were under the genetic control by the *Runx2* (that in our study was also overexpressed in 2DF-iMacs) [48]. Thus, our data on transcriptional priming of 2DF-iMacs for cholesterol accumulation and M2-like activity are in line with published results and even extend them by demonstrating that unexperienced “naïve-like” M2-biased macrophages cultured in “normal” (i.e., LDL not-enriched) conditions may be already predisposed for cholesterol accumulation. 

As discussed above, in iMA-EBS-iMacs, the overexpression of inflammation-related genes coupled with a higher secretion of pro-inflammatory cytokines. Whether the overexpression of genes associated with antigen presentation and lipid homeostasis in 2DF-iMacs manifests itself at a functional level has not been examined in this study. mRNA and protein levels do not always correlate with each other due to post-transcriptional processing, mRNA stability and post-translational modifications [39]. Therefore, theoretically, the transcriptomic dissimilarities between EBS- and 2DF-iMacs may manifest themselves or have not as statistically significant functional differences between these cell populations. Nevertheless, given that multiple genes associated with antigen presentation and lipid homeostasis were differently expressed between EBS- and 2DF-iMacs, it is possible to conclude that the two types of iMacs differ by their predisposition to antigen presentation and lipid accumulation. In certain microenvironments, these predispositions may manifest themselves at a functional level, therefore it is important to take them into account when generating iMacs for different applications. 

Of note, arterial macrophages include two populations, one of which originates from yolk-sac-derived erythromyeloid precursors, while the other one is derived from blood monocytes [49,50,51,52]. The populations are supposed to play different roles in artery homeostasis and pathology, but their exact role in these processes is not yet clear, and it is difficult to study [53]. Given the differentiation of iMacs models of embryonic yolk sac hematopoiesis [25,54], iMacs and 2DF-iMacs in particular represent a valuable model for studying the role of embryonically derived human macrophages in atherogenic processes.

In addition to revealing the differences between mature EBS- and 2DF-iMacs, our study has also demonstrated inter-protocol differences in the dynamic transcriptome of cells differentiating into iMacs. Previously, flow cytometry and real-time PCR gene expression analyses showed that iMac differentiation goes through the formation of mesoderm (markers, CD140a and KDR), hemogenic endothelium (CD144 and CD34), hematopoietic progenitors (CD43 and CD241a/CD235a) and finally, myeloid iMac precursors (CD45, CD11b and CD14) [3,30,55]. The results of our study confirm these pathways at the transcriptional level and allow us to deepen our understanding of the processes ongoing during iMac differentiation. In particular, we have demonstrated that during 2DF differentiation, dynamic changes in the expression of genes associated with the above-mentioned differentiation stages occur faster and are more coordinated in time compared with EBS differentiation. Furthermore, iMac differentiation was accompanied by profound alterations in the expression of genes associated with general biological and developmental processes, including a down-regulation of genes implicated in DNA replication, translation and cell division and an up-regulation of genes associated with epithelial-to-mesenchymal transition, angiogenesis, coagulation and various signaling pathways. These general changes took place during both 2DF and EBS differentiations. In contrast, the dynamic expression of immunity/inflammation-related genes and genes involved in the control of lipid homeostasis differed between the protocols. Of note, these two gene expression categories were also differentially expressed in mature EBS- and 2DF-iMacs. One possible explanation for this coincidence could be that our dynamic RNA samples were contaminated with RNA derived from myeloid iMac precursors. However, this explanation seems to be unlikely, since in dynamic experiments, we intentionally removed floating cells (that contain iMac precursors) from the cultures before RNA isolation. Moreover, inter-protocol dynamic transcriptomic differences appeared as early as starting d_+6_, i.e., at a time point when myeloid iMac precursors were not yet generated. We conclude therefore that inter-protocol differences in cell inflammatory and lipid homeostasis pathways were fundamental. 

Comparing our dynamic transcriptomic data with published results is difficult due to a lack of the latter. Until recently, transcriptomic analyses of iMacs have mainly focused on their comparison with blood-monocyte-derived macrophages and the analysis of iMac response to inflammatory stimuli [3,5,40]. iMac differentiation trajectories have not been followed, to the best of our knowledge. In 2020, Monkley and co-authors [56] published the first results of transcriptomic analysis of differentiating iMacs. The authors reported a complete switch of iPSCs to cells expressing monocyte- and macrophage-specific gene expression profiles during the differentiation. However, the analysis was restricted to iPSCs, myeloid iMac precursors and mature iMacs only, and did not include intermediate differentiation stages. Most recently, Jo and co-authors [57] performed single-cell RNA sequencing of mature iMacs generated from human embryonic stem cells and iPSCs using a variant of the 2DF protocol. The authors reported a high degree of homogeneity of the iMac population. At the same time, it was found that iMacs were composed of several clusters, some of which contained DEGs (e.g., genes associated with cell division, DNA replication and mRNA splicing). Whether cluster composition of iMacs generated using different protocols is different is an interesting question to address in the future.

Currently, factors underlying inter-protocol differences in iMac biology and differentiation trajectories cannot be precisely defined, but several of them should be considered. First, 2DF differentiation relies on the use of multiple exogenous factors able to drive mesoderm development (BMP4, VEGFA and CHIR99021) and that are directly implicated in hematopoietic processes (SCF, IL-6, IL-3 and M-CSF). In contrast, EBS differentiation relies on a spontaneous formation of mesoderm and a promotion of cell differentiation in a hematopoietic direction by only two factors, IL-3 and M-CSF. The use of multiple hematopoiesis-related factors seems to be the major reason responsible for more rapid and coordinated dynamics of gene expression in the 2DF compared with the EBS protocol. 

Another significant inter-protocol difference is the use of different media at early differentiation stages (i.e., KO-DMEM, X-VIVO™15 and RPMI-1640 in EBS and mTeSR^TM^1, StemPro^TM^-34 and RPMI-1640 in 2DF protocols). The composition of most of the used media is not open, making their direct comparison impossible. However, it is clear that the media may differ by components and component concentrations, and that these differences may affect cell physiology. Vaughan-Jackson and co-authors [58] have recently performed a detailed side-by-side comparison of iMacs generated using the EBS protocol in X-VIVO 15 or home-made fully defined OXM medium based on the advanced DMEM-F12. The authors found significant transcriptional differences between the two iMac populations, in particular in the expression of genes associated with macrophage polarization/inflammatory potential and lipid metabolism, i.e., the two gene categories that in our study were different between EBS- and 2DF-iMacs. 

Among other methodological differences between the two protocols, cell cultivation in hypoxic conditions in 2DF and in normoxic conditions in EBS protocols should be mentioned. As recently shown, hypoxia is a physiological condition for embryonic hematopoiesis. In vitro, it enhanced endothelial-to-hematopoietic transition and the generation of hematopoietic progenitors from human iPSCs [59]. Thus, in our model, hypoxia could contribute to a clearer stage-wise transition of differentiating cells in 2DF culture conditions. 

Speaking about the choice of iMac differentiation protocol, several parameters are usually taken into account. EBS protocols are less expensive and are labor-intensive, which underlies their more frequent use in experimental studies. However, EBS protocols are feeder- and serum-dependent, whereas 2DF protocols have an advantage of using feeder-free (or feeder-depleted) conditions and chemically defined serum-free medium. In this study, we revealed other differences between the two types of protocols that need to be taken into account when choosing the method of iMac generation. First, we have documented that 2DF protocols induce a faster and a more coordinated transition of cells through the differentiation process, which is important for the reliability of iMac differentiation results. Second, we identified significant differences between the transcriptomic profiles of EBS- and 2DF-iMacs related to such important macrophage features as cell inflammatory potential, antigen presentation and lipid homeostasis. Based on these results, it can be assumed that the EBS protocol is preferable when an inflammatory macrophage response is desirable (e.g., when developing iMacs for the treatment of cancer or early stage infections), whereas the 2DF protocol has advantages when it is important to minimize inflammatory reactions or ensure effective antigen presentation (e.g., for the treatment of chronic infections), as well as for the modeling of foam macrophage formation and atherogenesis. Overall, the results indicate that different protocols may be optimal for generating iMacs that serve different purposes and that it is important to take this into account to facilitate advances in further iMac applications.

## 4. Materials and Methods

### 4.1. Reagents

The suppliers and catalogue numbers of all reagents are presented in Table S8.

### 4.2. iPSC Cell Lines

iPSC lines iMA and K7-4Lf were generated from human embryonic dermal fibroblasts and peripheral blood monocytes, respectively (a kind gift by Dr. E. Grigor’eva [33,34]). iPSCs were expanded and passaged on mouse embryonic fibroblast feeder cells (MEFs) as described earlier [32]. 

### 4.3. The Differentiation of iMacs Using the EBS Protocol

The generation of iMacs using the EBS protocol (EBS-iMacs) was performed as described earlier [32]. Briefly, iPSCs were expanded on MEFs, collected using collagenase IV in a way to preserve colony integrity, placed in ultralow-adhesive 6-well plates and cultured in supplemented KO-DMEM medium (15% KOSR, 2 µM Glutamax, 1% non-essential amino acids, 1% penicillin/streptomycin and 0.055 mM β-mercaptoethanol) to allow EB formation (day −4). Four days later (day 0), the generated EBs were harvested, transferred to tissue culture 6-well plates (15–20 EBs/well) and cultured in supplemented X-VIVO™15 medium (2 µM Glutamax, 1% penicillin/streptomycin and 0.055 mM β-mercaptoethanol) with the addition of IL-3 (25 ng/mL) and M-CSF (50 ng/mL). Full medium change was performed every 7 days. When floating monocytic precursors of iMacs appeared in the cultures, they were collected, transferred to new wells and cultured in supplemented RPMI-1640 medium (10% FCS, 2 µM L-Glutamax, 1% non-essential amino acids, 1% penicillin/streptomycin and 0.055 mM β-mercaptoethanol) containing M-CSF (50 ng/mL) to induce terminal differentiation of EBS-iMacs. The remaining cultures were restimulated with supplemented X-VIVO™15 medium containing IL3/M-CSF for another round of iMac generation (Figure 1a). 

### 4.4. The Differentiation of iMacs Using the 2DF Protocol

The generation of iMacs using the 2DF protocol (2DF-iMacs) was performed as it was previously described by Takata and co-authors [29], with some modifications. Briefly, iPSCs were expanded on MEFs and depleted from MEFs by culturing them on Matrigel-coated tissue culture plates in mTeSRTM1 medium for 2–3 days. MEF-depleted iPSC colonies were collected using collagenase IV and transferred to new Matrigel-treated tissue culture 6-well plates at a 1:10–1:12 ratio (day −6). The cells were then cultured in StemProTM-34 medium that was supplemented with mixtures of factors stimulating mesoderm/hemogenic endothelium (day −6 to day 0) and hematopoietic/myeloid differentiation (day 0 to day +10). The medium was changed every other day. On day +10, StemProTM-34 was replaced by supplemented RPMI-1640 medium containing M-CSF (50 ng/mL). The medium was changed every 3 days. When the first 2DF-iMacs appeared in the cultures (which usually occurred around day +19), they were collected and used for phenotypic and functional analysis or RNA isolation. In the protocol by Takata and co-authors [29], the remaining cultures were terminated after the first iMac harvesting. In our modification of the protocol, the remaining cultures were re-stimulated with supplemented X-VIVO™15 medium containing IL-3 (25 ng/mL) and M-CSF (50 ng/mL). Seven days later, monocyte-like precursors of 2DF-iMacs appeared in the cultures; they were collected, transferred to new 12-well or 6-well plates and cultured in supplemented RPMI-1640 medium containing M-CSF (50 ng/mL) for 7 days to obtain a new portion of 2DF-iMacs. The rounds of iMac generation were repeated multiple times while cell generation lasted (usually for more than 90 days). Morphological and phenotypic analyses showed similarities of 2DF-iMacs generated using the original and modified 2DF protocols (not shown). Culture conditions were hypoxic from day −6 to day +2 and normoxic starting day +2 (Figure 1b). 

### 4.5. Cell Morphology, Cytospin Preparation and Staining

Light microscopy images were obtained using an ADF 1350B microscope equipped with an ADF Live 4h camera (ADF optics Co., Ltd., Jiaxing, China). Cytospins were prepared using a Shandon cytocentrifuge (Thermo Scientific, Langenselbold, Germany). Briefly, 3–4 × 10^3^ cells were spun for 3 min at 1000 rpm. Slides were dried, treated with methanol for 5–10 min, stained with Romanovsky–Giemsa stain (Merck, Burlington, MA, USA) for 15–30 min, dried and analyzed using bright-field microscopy.

### 4.6. Flow Cytometry and Cell Sorting

Cells (2–3 × 10^5^/sample) were stained with antibodies (see Table S8), washed, fixed and analyzed on a Cytoflex-S (Beckman Coulter, Brea, CA, USA) or Attune^TM^ NxT (Thermo Fisher Scientific, Whatham, MA, USA) flow cytometer using CytExpert^TM^ (Beckman Coulter), Invitrogen™ Attune™ NxT (Thermo Fisher Scientific) and FlowJo (TreeStar BD Bioscience, Franklin Lakes, NJ, USA) software. EBS-iMacs and 2DF-iMacs were differentiated in parallel and were stained and analyzed simultaneously using the same instrument settings. For instrument setup, unstained and single-stained samples were used. Isotype controls were performed in preliminary experiments to check the levels of non-specific antibody binding (which were insignificant). Preliminary experiments showed that cell staining with anti-CD14 antibodies widened the negative population similar to fluorescence—minus one control. Therefore, in all experiments, samples stained with anti-CD14 antibodies were used as gating control. To obtain pure iMac populations, terminally differentiated EBS-iMacs and 2DF-iMacs were stained with PerCP-anti-CD14 antibodies and FACS-sorted using S3E (Bio-Rad, Hercules, CA, USA) or FACS Aria™ Fusion (BD Bioscience, Franklin Lakes, NJ, USA) cell sorters.

### 4.7. iMac Phagocytic Activity 

Phagocytosis was assessed using the commercial Phagotest™ kit (BD Bioscience); all procedures were performed according to the manufacturer’s instructions as described earlier [32].

### 4.8. Multiplex Analysis

The differentiated EBS-iMacs and 2DF-iMacs were cultured in a 24-well plate (2 × 10^5^ cells/well) and either stimulated with LPS (100 ng/mL) and recombinant human IFN-γ (20 ng/mL) or left unstimulated. The supernatants were collected 24 h later and analyzed using a 41-pex MILLIPLEX MAP Human Cytokine/Chemokine kit that detects EGF, CCL11, CSF2, CSF3, IFNα2, IFNγ, IL-10, IL-12P40, IL-12P70, IL-13, IL-15, IL-17A, IL-1RA, IL-1α, IL-1β, IL-2, IL-3, IL-4, IL-5, IL-6, IL-7, CXCL8, CXCL10, CCL2, CCL3, CCL4, CCL5, TNFα, TNFβ, VEGF, FGF-2, TGF-α, FLT-3L, CXC3CL1, CXCL1, CCL7, CCL22, PDGF-AA, PDGF-AB/BB, sCD40L and IL-9. All the procedures were performed in accordance with the manufacturer’s recommendations.

### 4.9. RNA Isolation, Transcriptome Library Preparation and Sequencing

To compare the transcriptomic profiles and the differentiation trajectories of EBS-iMacs and 2DF-iMacs, the same iPSCs were differentiated into iMacs in parallel, using EBS and 2DF protocols. In EBS and 2DF protocols, it takes different time intervals to differentiate iPSCs into mesoderm, i.e., 4 and 6 days, respectively. To account for the differences, day 0 was taken as the day when mesoderm is formed and hematopoietic specification is initiated. RNA was isolated from the following: (i) iPSCs just before the start of iMac differentiation (day −4 in EBS and day −6 in 2DF protocol); (ii) differentiating cells at different differentiation time points (days 0, +6, +10 and +19); (iii) FACS-sorted CD14+ EBS-iMacs and 2DF-iMacs (for RNA-seq, first harvests of iMacs were used). For RNA isolation, the cells were homogenized using syringe and needle or a QIAshredder kit, and RNA was isolated using RNeasy Micro (<5 × 10^5^ cells) or RNAeasy Mini (>5 × 10^5^ cells) Kits (Qiagen, Germantown, MA, USA). 

RNA concentrations were measured using a Qubit™ RNA HS Assay Kit and RNA integrity number (RIN) was assessed using an RNA 6000 Pico Kit for Bioanalyzer. Only RNA samples with RIN > 8 were used for subsequent library preparation. PolyA RNA was isolated using a NEBNext Poly(A) mRNA Magnetic Isolation Module, containing magnetic Oligo d(T) beads. Transcriptome libraries were constructed using a NEBNext Ultra II RNA Library Prep Kit for Illumina following manufacturer’s instructions. Libraries were prepared with a starting amount of 150 ng and amplified with 15 PCR cycles. RNA was fragmented to the average size of 200 nt by incubation at 94 °C for 10 min. Libraries were quantified with a Qubit dsDNA HS Kit and the size distribution was determined with a High Sensitivity DNA Kit for Bioanalyzer. Libraries were ~ 350 bp in length; they were sequenced on Illumina HiSeq 4000 (Illumina, San Diego, CA, USA) in single read mode with the read length of 51 nt and on Illumina NextSeq 500 (Illumina, San Diego, USA) in single read mode with the read length of 76 nt. 

### 4.10. RNA-Seq Data Analysis and Bioinformatics

CLC Genomics Workbench 20.0.4 (https://digitalinsights.qiagen.com, accessed on 5 September 2022) was used for read trimming with the following parameters: “quality scores—0.005; trim ambiguous nucleotides—2; remove 5′-terminal nucleotides—1; remove 3′-terminal nucleotides—1; discard reads below a length of 25”. The high-quality reads were uniquely mapped using the CLC Genomics Workbench (length fraction = 1 and similarity fraction = 0.95) to the reference genome of H. sapiens (GRCh38 version). Differential expression analysis was performed with “DESeq2” R package [36]. FDR < 0.05 and log2 FC > |1| were used to select differentially expressed genes. Time-series analyses in which gene expression in two protocols was compared in dynamics were conducted using three different approaches. Firstly, DEGs were identified between consequent stages of iMacs differentiation for 2DF and EBS protocols separately. Two replicates of iMA trajectories and one replicate of K7 were used together. DEGs were identified using “DESeq2”. Secondly, genes with differences in dynamics between protocol of differentiation were identified via “DESeq2” and design formula “~cellType + protocol + time + protocol:time”, where “cellType” stands for iPSC lines (iMA or K7), “protocol” corresponds to the differentiation protocol (EBS or 2DF), and “time” means time points. Finally, pathway-level analysis was performed by gene set co-regulation analysis (GESECA) implemented in “fgsea” R package [60]. For this analysis, the expressions of replicates were averaged allowing the comparison of equal length time-series data vectors with a single value for each time point (day −6/−4, day 0, day +6, day +10 and day +19). Human Hallmark gene sets (also referred to as pathways) from the Molecular Signatures Database (MSigDB) [61,62] were obtained by “msigdbr” R package [63]. The four experiments (i.e., iMA-EBS, iMA-2DF, K7-EBS and K7-2DF) were analyzed separately with “geseca” function to estimate the impact of each gene set on the variability of gene expression in the time-series data (pctVar). Results not passing the threshold for an adjusted *p*-value (padj < 0.05) were filtered out and the remaining pathways were visualized with “plotGesecaTable” function.

Heatmaps were generated with a “complexHeatmap” R package [64]. Testing for functional enrichment of differentially expressed genes (DEGs) was performed using the Database for Annotation, Visualization, and Integrated Discovery (DAVID) online tool [65]. The categories analyzed included GO terms [65], KEGG pathways [65] and UP-KW [66]. The Benjamini–Hochberg (BH) correction for multiple testing was performed with a cutoff for an adjusted *p*-value of < 0.05. 

To construct clusters of inter-protocol DEGs, the STRING (version 9.1) was used. The input options were set to include a confidence cutoff 0.4 and maximum additional interactions 0. The construction of protein–protein interaction networks and the functional enrichment of clusters were performed in Cytoscape 3.9.1 [67].

### 4.11. Statistical Analysis

Most data are shown as medians and interquartile ranges (25%; 75%); differences between the groups were analyzed using the non-parametric Kruskal–Wallis and post hoc Mann–Whitney tests (GraphPad Software Inc., San Diego, CA, USA). Normally distributed data (Phagotest results) are shown as mean ± SD (95% confidence interval). For multiple group comparisons (phenotypic markers and multiplex assay) and GO pathway analysis, FDR was controlled using the BH method with FDR set at q = 0.05 [68] (R-studio, R-Tools Technology, Richmond Hill, ON, Canada). 

## Figures and Tables

**Table 1:** Categories from Gene Ontology and other databases over-represented in iMA- and K7-derived EBS-iMacs and 2DF-iMacs *.

Comparison	Pattern (DEG Number)	Biological Process/ Pathway Category	Source	DEG Number	FC	FDR	Identity of DEGs
iMA- EBS-iMacs versus iMA- 2DF-iMacs	Up-regulated in EBS-iMacs (49) *	cellular response to interleukin-1	GOTERM_BP_DIRECT	5	10.2	2.3 × 10^−2^	*CCL2, CCL8, MYC, CHI3L1* and *ICAM1*
		cellular response to tumor necrosis factor	GOTERM_BP_DIRECT	6	15.7	2.3 × 10^−2^	*CCL2, CCL8, GATA3, CHI3L1, ICAM1* and *POSTN*
		inflammatory response	GOTERM_BP_DIRECT	8	7.2	2.3 × 10^−2^	*BCL6, CCL2, CCL8, NLRP2, CHI3L1, FASN, IL1RN* and *PTGER2*
		inflammatory response	UP_KW_BIOLOGICAL_ PROCESS	5	9.6	4.6 × 10^−2^	*BCL6, CCL2, CCL8, NLRP2* and *CHI3*
	Up-regulated in 2DF-iMacs (118) *	ZN_FING:C2H2-type 10	UP_SEQ_FEATURE	15	6.7	4.1 × 10^−6^	*ZNF117, ZNF253, ZNF287, ZNF329, ZNF331, ZNF347, ZNF 404, ZNF415, ZNF471, ZNF486, ZNF568, ZNF595, ZNF626, ZNF677* and *ZNF93*
		Herpes simplex virus 1 infection	KEGG_PATHWAY	13	3.6	2.0 × 10^−2^	*HLA-F, HLA-DRB1, ZNF253, ZNF331, ZNF347, ZNF404, ZNF415, ZNF471, ZNF486, ZNF568, ZNF595, ZNF677* and *ZNF93*
		RNA polymerase II transcription factor activity and sequence-specific DNA binding	GOTERM_MF_DIRECT	23	3.3	1.6 × 10^−4^	*ELF3, SOX4, TBX15, ATOH8, PAX8, TSHZ3, ZSCAN18, ZNF117, ZNF253, ZNF287, ZNF329, ZNF331, ZNF347, ZNF404, ZNF415, ZNF471, ZNF486, ZNF568, ZNF595, ZNF626, ZNF677, ZNF844* and *ZNF93*
K7- EBS-iMacs versus K7- 2DF-iMacs	Up-regulated in K7-iMacs (126) *	endocytic vesicle membrane	GOTERM_CC_DIRECT	8	15.5	1.4 × 10^−4^	*CD74, CD9, WNT5A, CSF3R, HBEGF, MSR1, HLA-DPA1* and *HLA-DQB1*
		MHC class II protein complex	GOTERM_CC_DIRECT	4	30.4	6.3 × 10^−3^	*CD74, HLA-DMB, HLA-DPA1* and *HLA-DQB1*
		antigen processing and presentation of exogenous peptide antigen via MHC class II	GOTERM_BP_DIRECT	5	26.9	3.2 × 10^−2^	*CD74, FCGR2B, HLA-DMB, HLA-DPA1* and *HLA-DQB1*

* Other over-represented categories are presented in Table S1.

## Data Availability

Access to RNA-seq datasets is provided in the Sequence Read Archive (SRA, NCBI) repository under accession PRJNA893554 and the Gene Expression Omnibus (GEO, NCBI) repository under accession GSE220450. The data are also available in Table S7.

## Supplementary Materials

The supporting information can be downloaded at: https://www.mdpi.com/article/10.3390/ijms232416087/s1.
